# Intradetrusor OnabotulinumtoxinA Injections Ameliorate Autonomic Dysreflexia while Improving Lower Urinary Tract Function and Urinary Incontinence-Related Quality of Life in Individuals with Cervical and Upper Thoracic Spinal Cord Injury

**DOI:** 10.1089/neu.2020.7115

**Published:** 2020-08-27

**Authors:** Matthias Walter, Stephanie L. Kran, Andrea L. Ramirez, Daniel Rapoport, Mark K. Nigro, Lynn Stothers, Alex Kavanagh, Andrei V. Krassioukov

**Affiliations:** ^1^ International Collaboration on Repair Discoveries (ICORD), Faculty of Medicine, University of British Columbia, Vancouver, British Columbia, Canada.; ^2^ MD Undergraduate Program, Faculty of Medicine, University of British Columbia, Vancouver, British Columbia, Canada.; ^3^ Department of Urologic Sciences, Faculty of Medicine, University of British Columbia, Vancouver, British Columbia, Canada.; ^4^ Division of Physical Medicine and Rehabilitation, Faculty of Medicine, University of British Columbia, Vancouver, British Columbia, Canada.; ^5^ G.F. Strong Rehabilitation Centre, Vancouver, British Columbia, Canada.

**Keywords:** autonomic dysreflexia, complications, neurogenic detrusor overactivity, onabotulinumtoxinA, spinal cord injury

## Abstract

Pilot data of our phase IV clinical trial (pre/post study design) highlighted a beneficial effect of intradetrusor onabotulinumtoxinA (200 IU) injections to reduce autonomic dysreflexia (AD) in individuals with chronic spinal cord injury (SCI) at T6 or above. After trial completion, we assessed whether our primary expectation (i.e., decrease of AD severity in 50% of participants during urodynamics [UDS]) was met. Secondary outcome measures were reduction of spontaneous AD in daily life as well as amelioration of AD-related and urinary incontinence-related quality of life (QoL). In addition, we conducted injury-level–dependent analysis—i.e., cervical and upper thoracic—to explore group-specific treatment efficacy. Post-treatment, AD severity decreased in 82% (28/34) of all participants during UDS and in 74% (25/34) in daily life assessed with 24-h ambulatory blood pressure monitoring. In addition, urinary incontinence-related QoL was improved, cystometric capacity was increased, and maximum detrusor pressure during storage was reduced (all *p* < 0.001). Further, the treatment was well tolerated, with only minor complications (grade I [*n* = 7] and II [*n* = 7]) in accordance with the Clavien-Dindo classification recorded in 11 individuals (cervical *n* = 9, upper thoracic *n* = 2). Injury-level–dependent analysis revealed lower incidence (cervical *n* = 15/23, upper thoracic *n* = 6/11) and lesser severity (cervical *p* = 0.009; upper thoracic *p* = 0.06 [Pearson *r* = −0.6, i.e., large effect size]) of AD during UDS. Further, reduced AD severity in daily life, improved urinary incontinence-related QoL, greater cystometric capacity, and lower maximum detrusor pressure during storage (all *p* < 0.05) were found in both groups post-treatment. Intradetrusor onabotulinumtoxinA injections are an effective and safe second-line treatment option that ameliorates AD while improving lower urinary tract function and urinary incontinence-related QoL in individuals with cervical and upper thoracic SCI.

## Introduction

Besides the loss of sensorimotor function, impairment of autonomic function ranks among the highest priorities for individuals after spinal cord injury (SCI).^1^ As such, neurogenic detrusor overactivity (NDO) and autonomic dysreflexia (AD) combine to place a tremendous burden on health and quality of life (QoL) in this population.

We have shown previously that the presence of NDO and the neurological level of injury (NLI) are independent risk factors for AD during urodynamics (UDS).^2^ Considering the latter, the higher the NLI above T6, the higher the odds of experiencing AD, thereby leaving those with cervical SCI at the highest health risk.^2^ A single episode of AD can result in potentially life-threatening complications (i.e., stroke and myocardial infarction or even death); therefore, urologists are advised to take precautions when conducting UDS in this population.^3^

OnabotulinumtoxinA has been shown to improve effectively lower urinary tract (LUT) function in this population that includes the reduction or complete elimination of NDO.^4^ Our pilot data comprising 17 individuals with SCI (i.e., NLI at or above T6) confirmed the former but also suggested a beneficial effect of onabotulinumtoxinA to ameliorate AD.^5^ The primary end-point of this trial was a decrease of severity of AD in 50% of participants after intradetrusor onabotulinumtoxinA injections. The secondary end-points were a reduction of spontaneous AD during daily living assessed with 24-h ambulatory blood pressure monitoring (24-h ABPM), amelioration of AD-related QoL, and incontinence-related QoL compared with baseline.

We present the findings of our entire cohort after trial completion. In addition, we conducted an injury-level–dependent analysis to investigate the efficacy of intradetrusor onabotulinumtoxinA injections in individuals with a cervical (c-SCI) and upper thoracic (T1 to T6) injury (ut-SCI).

## Methods

This prospective phase IV clinical trial using a pre-post study design was approved by the University of British Columbia Clinical Research Ethics Board and registered at clinicaltrials.gov (identifier NCT02298660). Fifty-five individuals with chronic SCI (>1-year post-injury) at T6 were screened (flow diagram shown in Supplementary Fig. S1) according to inclusion and exclusion criteria.^5^ After providing written informed consent according to the Helsinki II declaration, 49 individuals were assigned to a battery of assessments for eligibility. The NLI and completeness of SCI were classified according to the International Standards for Neurological Classification of SCI.^6^

All UDS were performed in accordance with the International Continence Society to document the extent of neurogenic LUT dysfunction at baseline.^7^ Cardiovascular parameters, such as systolic blood pressure (SBP), were recorded during UDS to reveal the presence of AD^8^ and its severity (i.e., change in SBP from baseline). Further, frequency and severity of AD-related symptoms as well as AD in daily life (i.e., 24-h ABPM^9^) were recorded. In addition, urinary incontinence-related QoL was assessed using the validated, standardized I-QoL questionnaire.^10^

Forty-five individuals with chronic SCI at T6 or above with a confirmed history of AD and NDO were included and assigned to undergo intradetrusor onabotulinumtoxinA injections (200 IU) intended to improve LUT function and ameliorate bladder-related AD. One month after intradetrusor onabotulinumtoxinA injections, UDS, 24-h ABPM, and questionnaires were repeated to assess treatment efficacy. Surgical complications were recorded until one month post-surgery using the Clavien-Dindo classification.^11^ Statistical analysis was performed using R Studio (Version 1.1.456). Using non-parametric statistics (i.e., Wilcoxon signed-rank test), data are presented as medians with interquartile ranges.

## Results

In total, 34 individuals (eight females, median age 44 years [36–48] and median time post-injury 14 years [5–22]; for individual data, see Supplementary Table S1) completed the study and were included in the overall and injury-level–dependent analyses—that is, c-SCI (*n* = 23) and ut-SCI (*n* = 11). The majority had a motor-complete SCI in accordance with the American Spinal Injury Association impairment scale (AIS; A = 19, B = 11, C = 3, or D = 1).^6^

Considering the small number of individuals with ut-SCI, we further calculated the effect size, expressed as Pearson correlation coefficient—i.e., Pearson (*r*), that is Z statistics divided by square root of total number of pairs in accordance with Rosenthal.^12^ Pearson *r* can vary in magnitude from −1 to 1, with −1 indicating a perfect negative linear relation, 1 indicating a perfect positive linear relation, and 0 indicating no linear relation between two variables (effect sizes: small, *r* = 0.1–0.29 or −0.1–(−0.29); medium, *r* = 0.3–0.49 or −0.3–(−0.49); large, *r* = 0.5 and greater or −0.5 and smaller).

The AD severity decreased in 82% (28/34) of all participants during UDS, which exceeded our expectations (i.e., the primary outcome measure was a decrease of AD severity in 50% of participants). The AD severity in daily life decreased in 74% (25/34) of all participants (i.e., main secondary outcome measure). Post-treatment, LUT function was significantly improved overall and group-specifically.

**FIG. 1.**
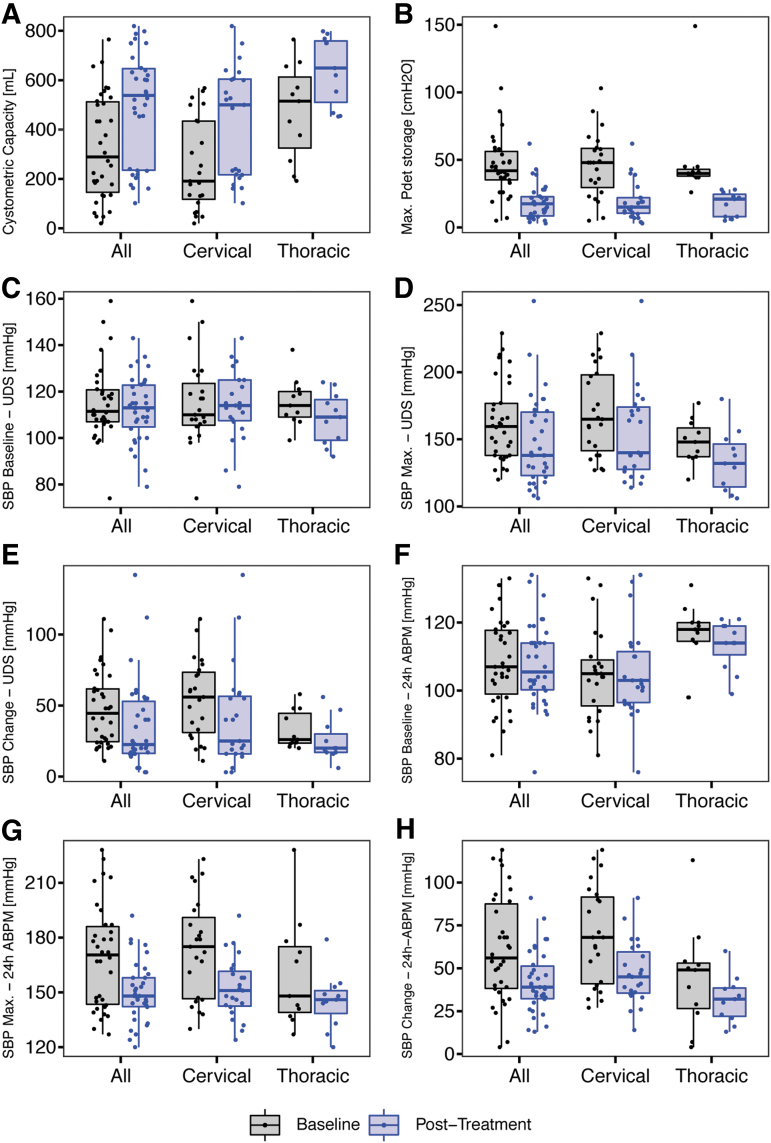
Pre/post-treatment comparisons of lower urinary tract function, cardiovascular changes during urodynamics (UDS) and in daily life, and quality of life related to autonomic dysreflexia (AD) symptoms and incontinence across all participants and injury-level–dependent subgroups. Compared with the initial assessment, we observed a variety of changes post-treatment: (**A**) cystometric capacity, (**B**) maximum detrusor pressure, (**C**) baseline systolic blood pressure (SBP) at the beginning of UDS, (**D**) maximum SBP during UDS, (**E**) severity of AD during UDS (i.e., maximum change in SBP), (**F**) baseline SBP in daily life (i.e., 24-h ambulatory blood pressure monitoring [24-h ABPM]),(**G**) maximum SBP in daily life, and (**H**) severity of AD in daily life (i.e., maximum change in SBP observed during 24-h ABPM). Data are presented at group level using box plots (median, interquartile range) and individually (dots).

Cystometric capacity (Fig. 1A) significantly increased overall (290 mL [146–512] vs. 538 mL [236 − 647], *p* < 0.001) in individuals with cervical (c-SCI, 191 mL [118–434] vs. 500 mL [217–604], *p* < 0.001), and upper thoracic SCI (ut-SCI, 515 mL [325–613] vs. 649 mL [510–759], *p* = 0.007, Z = 3, *r* = 0.9). Maximum detrusor pressure (Fig. 1B) was significantly lower overall (42 cm H_2_O [35–56] vs. 18 cm H_2_O [8–23], *p* < 0.001), in c-SCI (48 cm H_2_O [30–58] vs. 15 cm H_2_O [10–22], *p* < 0.001), and ut-SCI (40 cm H_2_O [38–43] vs. 21 cm H_2_O [8–24.5], p = 0.006, Z = -3, *r* = -0.9).

Baseline SBP at the beginning of UDS (Fig. 1C) did not significantly change—i.e., overall (112 mm Hg [107–121] vs. 113 mm Hg [105–123], *p* = 0.6), in c-SCI (110 mm Hg [106–124] vs. 114 mm Hg [108–125], *p* = 0.9), or in ut-SCI (114 mm Hg [109–120] vs. 109 mm Hg [99–116], *p* = 0.2, Z = -1, *r* = -0.3). Maximum SBP during UDS (Fig. 1D) was significantly reduced overall (160 mm Hg [138–177] vs. 138 mm Hg [123–170], *p* = 0.007) and in c-SCI (165 mm Hg [142–198] vs. 140 mm Hg [128–174], *p* = 0.04). Although reduction in maximum SBP during UDS in ut-SCI (148 mm Hg [137–158] vs. 132 mm Hg [114–146], *p* = 0.06) did not reach statistical significance, we found a large effect size (Z = -2, *r* = -0.6).

Severity of AD during UDS (i.e., maximum change in SBP, Fig. 1E) was significantly reduced overall (44 mm Hg [24–62] vs. 22 mm Hg [16–53], *p* < 0.001) and in c-SCI (56 mm Hg [31–74] vs. 25 mm Hg [16–56], *p* = 0.009). Although improvements in ut-SCI (26 mm Hg [24–44] vs. 20 mm Hg [17–30], *p* = 0.06) did not reach statistical significance, we found a large effect size (Z = -2, *r* = -0.6) indicating a meaningful reduction in SBP (i.e., by 23%).

Baseline SBP in daily life—i.e., 24-h ABPM (Fig. 1F)—did not change significantly—i.e., overall (107 mm Hg [99–118] vs. 106 mm Hg [100–114], *p* = 0.7), in c-SCI (105 mm Hg [96–109] vs. 103 mm Hg [96–112], *p* = 0.9), or in ut-SCI (118 mm Hg [114–120] vs. 114 mm Hg [110–119], *p* = 0.3, Z = -0.8 *r* = -0.24). Maximum SBP in daily life (Fig. 1G) was reduced significantly overall (170 mm Hg [144–186] vs. 148 mm Hg [142–158], *p* < 0.001), in c-SCI (175 mm Hg [146– 91] vs. 151 mm Hg [142–162], *p* = 0.002), and in ut-SCI (148 mm Hg [139–175] vs. 146 mm Hg [138–151], *p* = 0.05, Z = -2, *r* = -0.6).

Severity of AD in daily life (i.e., maximum change in SBP observed during 24-h ABPM, Fig. 1H) was reduced significantly overall (56 mm Hg [38–88] vs. 39 mm Hg [32–51], *p* = 0.001) and in c-SCI (68 mm Hg [41–92] vs. 45 mm Hg [36–60], *p* = 0.006). Although improvements in ut-SCI (49 mm Hg [26–53] vs. 32 mm Hg [22–38] *p* = 0.08) did not reach statistical significance, we found a large effect size (Z = -2, *r* = -0.6) indicating a meaningful reduction in SBP by 17 mm Hg (i.e., 35%).

In addition, frequency of AD episodes (i.e., total and bladder-related), frequency and severity of the AD symptom scores, and urinary incontinence-related QoL (i.e., total within each subcategory) were ameliorated post-treatment (see Table 1 and Supplementary Fig. S2 for subgroup and individual data, respectively). Only minor complications (grade I [*n* = 7] and II [*n* = 7]) in accordance with the Clavien-Dindo classification were recorded in less than one-third of all participants (11/34, 32%), of whom the majority had c-SCI (9/11, 85%).

**Table 1.** Post-Treatment Change of Subjective Measures

Parameter	All (*n* = 34)			Cervical (*n* = 23)			Thoracic (*n* = 11)		
	Pre	Post	p	Pre	Post	p	Pre	Post	p and (Z, r)
24-h ABPM									
Total AD episodes	10 [6–21]	8 [4–13]	0.03	11 [8–22]	9 [6–16]	0.2	7 [5–15]	3 [1–7]	0.06 (−2, −0.6)
Bladder-related AD episodes	4.5 [2–8.8]	2 [1–4.8]	0.01	6 [1.5–10]	3 [1–5.5]	0.09	4 [2–6]	1 [0–2]	0.04 (−2, −0.6)
AD Symptoms									
Frequency score	10 [8–13]	6 [3–9.8]	<0.001	11 [8.5–13]	6 [3–9.5]	0.003	8 [5.5–13.5]	6 [3–9.5]	0.1 (−2, −0.6)
Severity score	7.5 [4.5–9]	4 [2–7.8]	<0.001	8 [6–9.5]	4 [2–7.5]	0.002	7 [3–11.5]	5 [1–7.5]	0.05 (−2, −0.6)
I-QoL									
Avoidance	56 [44–71]	78 [67–84]	<0.001	59 [46–70]	81 [69–84]	<0.001	56 [42–72]	75 [58–88]	0.006 (3, 0.9)
Psychosocial Impact	54 [32–82]	83 [65–96]	<0.001	44 [34–80]	83 [69–97]	<0.001	64 [38–86]	81 [54–92]	0.06 (2, 0.6)
Social embarrassment	42 [18–68]	72 [40–90]	<0.001	40 [12–58]	80 [35–90]	<0.001	45 [25–70]	65 [40–88]	0.004 (3, 0.9)
Total	50 [36–72]	78 [63–91]	<0.001	42 [39–72]	81 [63–91]	<0.001	58 [40–74]	73 [56–88]	0.01 (3, 0.9)

24-h ABPM, 24-hour ambulatory blood pressure monitoring; AD, autonomic dysreflexia; I-QoL, incontinence quality of life; *r* Pearson correlation coefficient; Z, Z-score.

Data are presented as medians with interquartile ranges (25%- 5% IQR).

## Discussion

In line with previous studies, which have been summarized recently in a systematic review and meta-analysis by Li and associates,^4^ we observed significant improvements of LUT function and urinary incontinence-related QoL, and well-known complications associated with intradetrusor onabotulinumtoxinA injections.^4^ Over the course of a one-month follow-up, however, we recorded far fewer urinary tract infections (*n* = 7, 20.6 vs. 50.2%) and less nausea (*n* = 1, 2.9 vs. 5.5%) and constipation (*n* = 1, 2.9 vs. 5.2%) but slightly more fatigue (*n* = 3, 8.8 vs. 5%), pain, and headache (*n* = 2, 5.9 vs. 4.6%) than reported previously.^4^

Analysis of our entire cohort (N = 34) confirmed our pilot data (*n* = 17) and thus is providing evidence for the amelioration of AD by lowering the incidence and severity of AD both during UDS and in daily life as well as reducing the symptoms of AD and improving urinary incontinence-related QoL as a consequence of the improvement in LUT function after intradetrusor onabotulinumtoxinA injections.

As an extension of our preliminary findings, injury-level–dependent analysis revealed efficacy of intradetrusor onabotulinumtoxinA injections to significantly ameliorate AD and its associated symptoms in individuals with c-SCl. Although similar post-treatment changes were seen in individuals with ut-SCI, statistical significance was not reached in five outcome parameters (*p* = 0.06 to *p* = 0.08), including maximum SBP and change in SBP during UDS, change in SBP in daily life, frequency of AD in daily life, and I-QoL subscale psychosocial impact. Further statistical analyses, however, revealed medium or large effect sizes in all of these five outcome parameters indicating meaningful post-treatment improvements despite the smaller number of participants with ut-SCI (*n* = 11) compared with those with c-SCI (*n* = 23).

## Conclusion

The findings from this phase IV clinical trial highlight that intradetrusor onabotulinumtoxinA injections are an effective and safe second-line treatment option to improve LUT function and urinary incontinence-related QoL, while ameliorating bladder-related AD in individuals with cervical and upper thoracic (T1 to 6) SCI. Considering the increased risk of cardiovascular disease in this cohort,^13^ these findings are crucial because AD can result in life-threatening consequences, jeopardizing the well-being and quality of life of affected individuals.

## Supplementary Material

Supplemental data

Supplemental data

Supplemental data
