# Supplementary figures and images for: Intradetrusor OnabotulinumtoxinA Injections Ameliorate Autonomic Dysreflexia while Improving Lower Urinary Tract Function and Urinary Incontinence-Related Quality of Life in Individuals with Cervical and Upper Thoracic Spinal Cord Injury
Source: J Neurotrauma. 2020 Aug 27;37(18):2023–7. doi: 10.1089/neu.2020.7115 (PMC7470218; doi:10.1089/neu.2020.7115)

## Supplementary Data

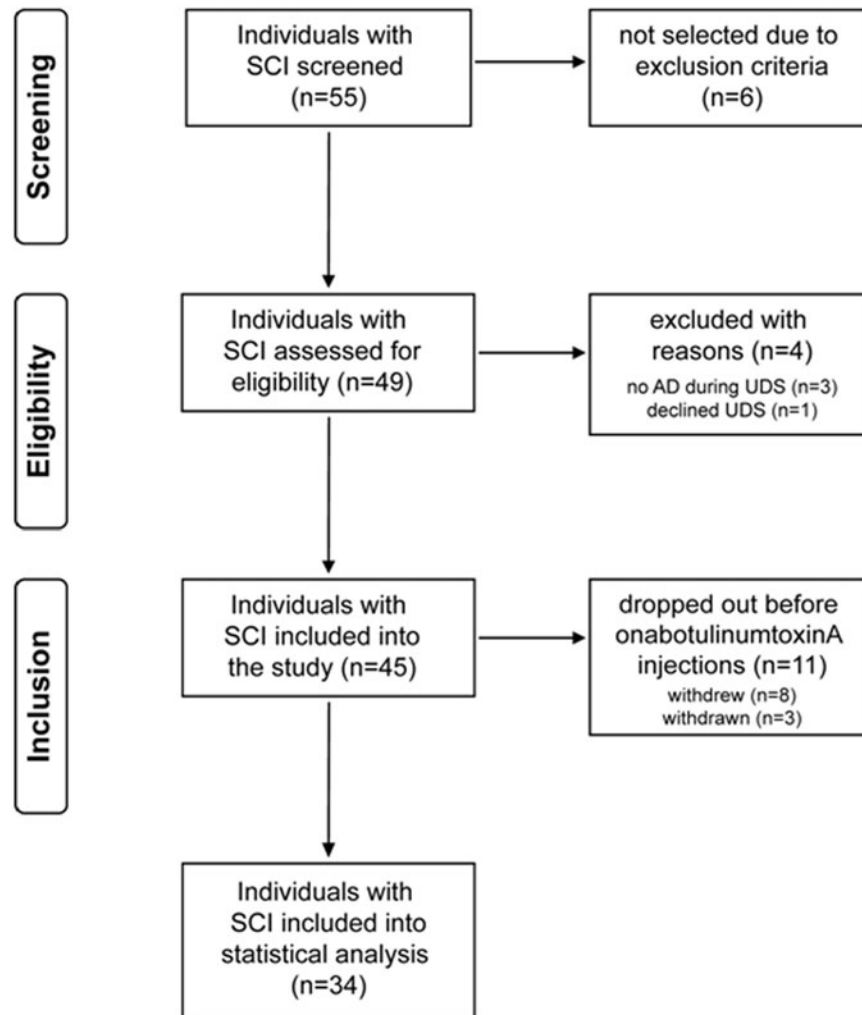

**SUPPLEMENTARY FIG. S1.** Study flow diagram.

Supplement: Supplemental data [file Supp_Fig1.pdf]
