# Supplementary material for: Intradetrusor OnabotulinumtoxinA Injections Ameliorate Autonomic Dysreflexia while Improving Lower Urinary Tract Function and Urinary Incontinence-Related Quality of Life in Individuals with Cervical and Upper Thoracic Spinal Cord Injury
Source: J Neurotrauma. 2020 Aug 27;37(18):2023–7. doi: 10.1089/neu.2020.7115 (PMC7470218; doi:10.1089/neu.2020.7115)
Supplement: Supplemental data [file Supp_Table1.pdf]

Supplementary Table S1. Demographics and Injury Characteristics

No.	*NLI*	*AIS*	*Sex*	*Age (years)*	*Time post-injury (years)*	*Cause of spinal cord injury*	*Bladder management*	*History of onabotulinum-toxinA injections*
1	C1	C	M	43	27	MVA	IC	Naive
2	C4	A	F	35	13	Other	IC	Naive
3	C4	A	F	51	6	MVA	IC	Naive
4	C4	B	M	22	5	Other	IC	Naive
5	C4	D	M	46	1	MVA	Foley*	Naive
6	C5	A	M	47	6	Other	IC	Naive
7	C5	A	M	36	1	Sports	IC	Naïve
8	C5	B	M	31	2	Fall	IC	Naive
9	C5	B	M	31	2	Sports	IC	Yes (Once)
10	C5	B	M	46	30	Sports	Foley*	Naïve
11	C5	C	M	43	23	Sports	IC	Naive
12	C5	C	M	62	4	Fall	IC	Naive
13	C6	A	M	44	19	Sports	IC	Naïve
14	C6	A	M	59	32	MVA	IC	Naive
15	C6	A	F	43	14	MVA	IC	Naive
16	C6	B	F	55	20	Fall	IC	Naive
17	C6	B	F	34	15	MVA	Foley*	Naive
18	C7	A	F	42	18	MVA	IC	Naive
19	C7	B	M	26	7	MVA	IC	Naive
20	C7	B	M	36	4	Other	IC	Naive
21	C7	B	M	49	6	Sports	IC	Naive
22	C8	A	M	42	22	MVA	IC	Naive
23	C8	B	M	45	40	MVA	IC	Naive
24	T2	A	M	36	8	MVA	IC	Yes (Thrice)
25	T3	B	M	38	20	MVA	IC	Yes (Once)
26	T3	A	M	53	40	Other	IC	Naive
27	T4	A	M	30	10	Sports	IC	Naive
28	T4	A	F	36	21	Sports	IC	Naive
29	T4	A	M	22	5	MVA	IC	Naive
30	T4	A	M	56	2	MVA	IC	Naive
31	T5	A	M	62	42	MVA	IC	Naive
32	T5	A	M	43	17	Sports	IC	Yes (Once)
33	T5	A	F	45	29	Fall	IC	Naive
34	T5	A	M	48	1	MVA	IC	Naive

NLI, neurologic level of injury; AIS, American Spinal Injury Association Impairment Scale; C, cervical; M, males; MVA, motor vehicle accidents; IC, intermittent catheterization; F, females; T, thoracic.

*Individuals relying on Foley catheters before study transferred to IC after onabotulinumtoxinA injections.
