# Supplementary material for: Intradetrusor OnabotulinumtoxinA Injections Ameliorate Autonomic Dysreflexia while Improving Lower Urinary Tract Function and Urinary Incontinence-Related Quality of Life in Individuals with Cervical and Upper Thoracic Spinal Cord Injury
Source: J Neurotrauma. 2020 Aug 27;37(18):2023–7. doi: 10.1089/neu.2020.7115 (PMC7470218; doi:10.1089/neu.2020.7115)
Supplement: Supplemental data [file Supp_Fig2.pdf]

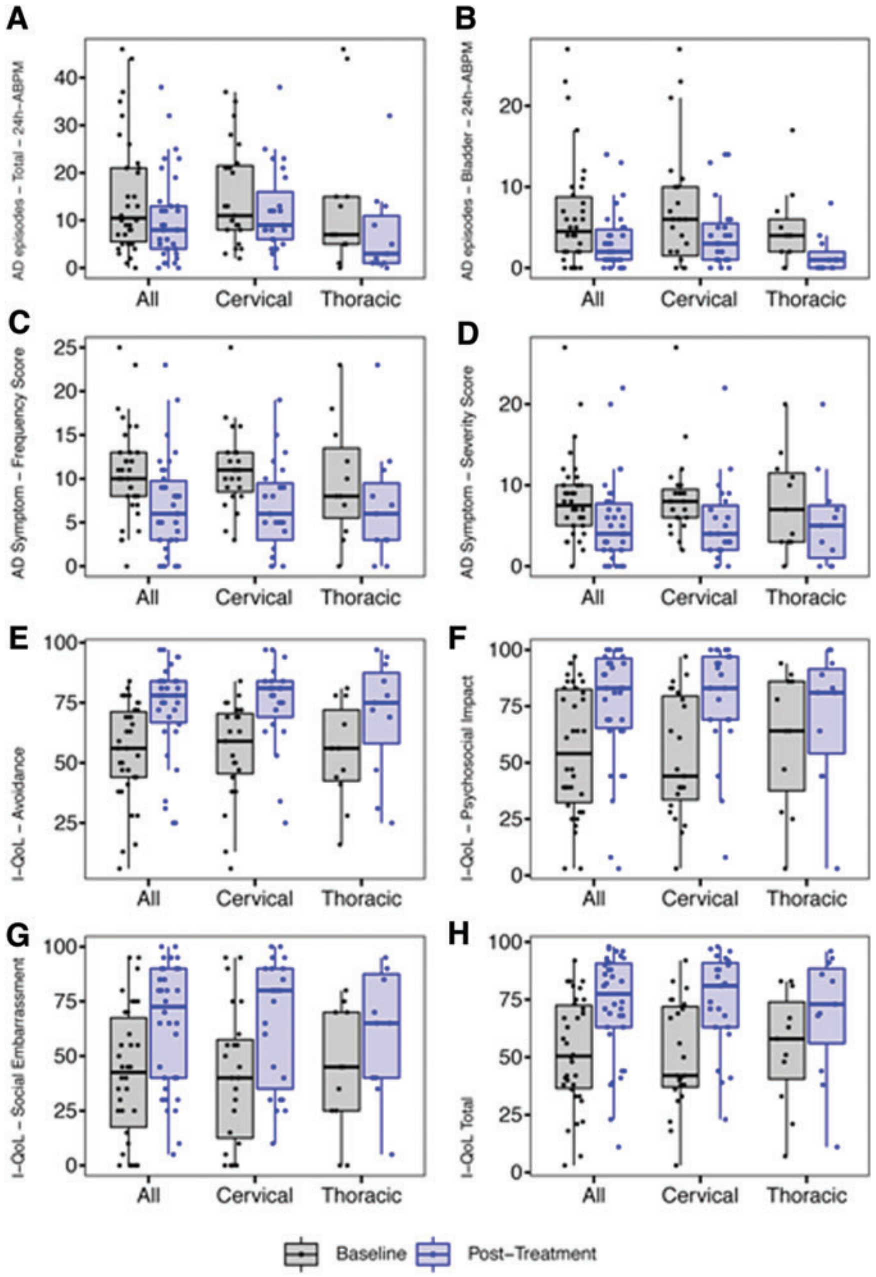

SUPPLEMENTARY FIG. S2. (**A**) Frequency of total autonomic dysreflexia (AD) episodes in daily life. (**B**) Frequency of bladder-related AD episodes in daily life. (**C**) AD symptoms frequency score. (**D**) AD symptoms severity score. Urinary incontinence-related quality of life (I-QoL)—subsections (**E**) avoidance and limiting behavior, (**F**) psychosocial Impact, (**G**) social embarrassment, and (**H**) in total. Data are presented at group level using box plots (median, interquartile range) and individually (dots).
